# Supplementary material for: Effectiveness and Cost-Effectiveness of Emergency Department–Based Violence Intervention Programs in the United Kingdom: Protocol for a Quasi-Experimental Study
Source: JMIR Res Protoc. 2026 Feb 18;15:e86247. doi: 10.2196/86247 (PMC12961390; doi:10.2196/86247)
Supplement: Multimedia Appendix 1 [file resprot_v15i1e86247_app1.docx]

The Template for Intervention Description and Replication (TIDieR)

| **Item** | **Description** |
| --- | --- |
| **Brief name** | *Provide the name or a phrase that describes the intervention.*  Hospital-based, clinically led Violence Prevention Team (VPT) |
| **Voice** | *"Who was involved in the preparation of TIDieR, how they were involved in the intervention/their perspective (e.g., researcher, service deliverer, patient, etc*  **Research and evaluation team**:  Wales Violence Prevention Unit (VPU) evidenced the need for the VPT and funded early implementation.  University evaluation team - Cardiff University (Violence Research Group and DECIPHER), Swansea University (SAIL Databank team), and York University contributed to the TIDieR.  Youth Endowment Fund (YEF) part-funded the intervention and funded the process and implementation evaluation. The National Institute for Health Research (NIHR134055) funded the effectiveness and cost-effectiveness evaluation.  An expert advisory group provides oversight to the evaluations, including experts from Public Health Wales, Trauma Network, South Wales Police, Office of the Police and Crime Commissioner (OPCC), and third sector organisations including Welsh Women’s Aid, The Wallich and Black Association of Women Step Out (BAWSO).  **Service Delivery**  VPT staff (nurse, nurse advocate, community youth workers), who implemented and continually develop the service.  Clinical teams in the two Emergency Departments (EDs) in which the interventions are situated provide clinical governance.  **Commissioners**  Wales VPU (partner agencies including South Wales Police OPCC, and Public Health Wales), provided the bulk of the funding for the interventions and have on-going dialogues regarding the service development, implementation, and delivery at both implementation sites.  The UK Home Office provided initial funding to the VPU and YEF |
| **Why** | *Describe any rationale, theory, or goal of the elements essential to the intervention.*  EDs receive for treatment patients who have been exposed to violence. There are numerous reasons why some people are exposed to violence, including their alcohol use, illicit drug use, unstable or chaotic homelife, amongst other reasons. These vulnerabilities will also be associated with increased utilisation of Emergency Care generally. EDs therefore have a unique opportunity to offer these patients additional support, beyond treatment for acute health needs, either directly or through referral to other healthcare services, or in discharge planning signposting third-sector organisations, for example. If this support is successful, then the expectation is that patients will exhibit a reduced use of emergency care services in general, not just for violence-related injury.  The primary objectives of the hospital-based service provision are to (i) identify patients attending EDs whose attendance is predicated on their exposure to violence, (ii) work with patients to understand any circumstances or vulnerabilities that increase their exposure to violence, and (iii) to either support their referral into secondary or third-sector care or to provide ongoing case-management alongside third-sector support.  All patient-facing clinical staff in emergency care, and elsewhere, have a duty of care and will have received training necessary to undertake patient safeguarding. However, the additional resources involved with the VPT afford (i) greater time working with patients to determine need, (ii) deepen links with primary, secondary, and tertiary care, and third sector organisations, for improved referral processes, (iii) work across clinical teams to support colleagues with their safeguarding needs and referral into the intervention, and (iv) through direct contact with patients, support opportunities for disclosure and therefore increase ascertainment of assault-related attendances.  While the VPT can and will refer to any community service provider based on patient need, specific funding has been dedicated to two different organisations where the VPTs are located. The community-based provision provides intensive support to high-risk children and young people (aged 11-24 years) involved in serious organised crime, drug-related activity, and showing signs of exploitation. The objective is to build resilience to enable these young people to be diverted away from further involvement in serious violence and organised crime. |
| **What - material** | *Describe any physical or informational materials used in the intervention, including those provided to participants or used in intervention delivery or in training of intervention providers. Provide information on where the materials can be accessed (e.g., online appendix, URL).*  The VPT have made resources available to patients and their families, including leaflets providing details of the service, and information packs to inform them of key issues (e.g., county lines and exploitation).  The VPT has available physical and informational materials usually available to ED staff. This includes access to patient records, both specific to the ED and community healthcare generally. This information facilitates risk assessment and safeguarding practices (e.g., identify patterns of attendance at health care settings for violent-related injury), as well as allowing the VPT ensure their engagement with patients is appropriate at that time (with consideration to the patient’s clinical needs).  In addition, the VPT have strong links with South Wales Police, and receive information and intelligence relating to violence related incidents and community problems. The provision of this information and intelligence to the intervention teams can help inform their interactions with patients and ensure the safety of the hospital and patients (e.g., in cases of youth violence where further attempts to harm a patient may occur).  **Training and support for staff by VPT**  The VPT provide training to a wide range of clinical and non-clinical staff within the hospital, including:  Clinical hospital staff, on violence (e.g., youth violence) and vulnerability, identifying violence-related injury and engaging with patients, implementing safeguarding procedures for violence-related injuries (e.g., completing multi-agency referral forms, MARFs).  Reception staff, on data entry and patient coding, to improve the quality of routine clinical data.  The nature of medical education entails short-term postgraduate and speciality training, and therefore a steady flow of clinical staff new to the ED environment. The VPT provides education and training, and impromptu advice and support through one-on-one interactions, to hospital staff. This includes providing consultation on patients and facilitating patient interactions for more challenging cases. |
| **What - procedures** | *Describe each of the procedures, activities, and/or processes used in the intervention, including any enabling or support activities.*  The VPT engages with patients at different stages of their journey, depending on the type and severity of their injuries, the time and day they attend the ED, and the longevity of their hospital stay. They are embedded in the ED clinical team, and have the same resources (e.g., access to electronic patient management systems) as other ED staff.  **Referral**  The VPT can receive patient referrals through multiple channels, including email, phone, and face-to-face contact. During their shift, VPT staff can be notified of eligible patients through a set of questions asked during patient registration, triage or by monitoring the ED patient management system. When the VPT is not on shift, paper-based referral forms are available, and staff can still use formats to refer patients and the VPT will retrospectively review the ED patient management system to identify any additional patients that may have been missed by clinical staff.  **Process**  The VPT provides individualised support to patients based on their needs and collaborates with primary, secondary and tertiary care, third sector organisations and other statutory organisations (e.g., local government safeguarding teams, the police, and school nurses). They establish a relationship with the patient and, where appropriate, their family. They provide emotional and practical support. They also manage vulnerabilities and risks to patients by gathering information and through collaborative multiagency work. This can be through existing resources, such as the Domestic Abuse, Stalking, Harassment and Honour Based Violence Assessment (DASH) tool, and referrals into the Multi Agency Risk Assessment Committee (MARAC).  The VPT will either discharge patients to receive support elsewhere, or, in the case of high risk, high need children exposed to criminal or sexual exploitation, continue their support of the patient alongside third sector organisations specifically funded to support the VPT in this area.  **Access to information**  The VPT has access to usual healthcare patient management systems, both ED specific and community-based, that can be accessed to inform patient assessments and safeguarding decisions.  Furthermore, the VPT access information on patients to inform risk assessment and management. For example, following incidents of serious violence (e.g., stabbings and shootings), they will gather information on risks and known associations through the police, VPU team (i.e., police and probation), hospital staff, and through the safeguarding team (who routinely meet to discuss patients).  **Assessment**  The VPT completes a risk- and needs-based assessment in collaboration with patients if they agree to engage and, where appropriate, their families. This will also include information obtained through patient records (e.g., PARIS and clinical portal) on previous hospital visit. |
| **5. Who provided** | *For each category of intervention provider (e.g., psychologist, nursing assistant), describe their expertise, background and any specific training given.*  VPT staff will be trained to Level 2 or Level 3 Safeguarding, available through continuing professional development to all frontline staff in the NHS. VPT staff are typically seconded from the broader ED team and will typically be nurse-led.  Additional third-sector support is funded to provide support to high risk, high need children. |
| **6. How** | *Describe the modes of delivery (e.g., face-to-face or by some other mechanism, such as internet or telephone) of the intervention and whether it was provided individually or in a group.*  The VPT team meets with patients face-to-face in the ED if they are on shift, or on hospital wards if the patient is admitted. If the team is not on shift, or receive referrals from Minor Injury Units, they will follow-up with a phone call and conduct an assessment. All contact is done an individual basis, or in collaboration with the family (for under 16-year-olds, or in cases the patient consents to family involvement).  Under usual safeguarding processes, the clinician who first receives any disclosure is expected to lead on the subsequent referral to the VPT, so that patients are not required to repeatedly describe circumstances they might find distressing. In this latter case, the VPT will support the patient’s referral. |
| **7. Where** | *Describe the type(s) of location(s) where the intervention occurred, including any necessary infrastructure or relevant features.*  The VPT are physically based in the ED. However, they can accept referrals from Minor Injury Units, and also provide support to patients on the wards if they are admitted following serious injury. The VPT also work with patients that are transferred from other health care facilities- which is particularly pertinent when the hospital is a major trauma centre, or trauma unit, and therefore accepts out-of-area patients. The VPT engage with other health care settings to facilitate transfers (e.g., provide information on known risks and vulnerabilities), and inform care planning. |
| **8. When and how much** | *Describe the number of times the intervention was delivered and over what period of time including the number of sessions, their schedule, and their duration, intensity, or dose.*  Patients will either engage with the VPT or will refuse support. If the latter, they will have been in contact with the VPT once. For patients who engage with the VPT, the frequency and duration of engagement with patients will be determined through the patient’s age, clinical need, and any underlying vulnerability.  *Hospital based VPT*  The VPT typically have one or two interactions with most patients, such as a phone call or text message. However, patients with greater needs may have more frequent contact and remain on the caseload for several weeks. Inpatients are supported until they are discharged. The VPT can only maintain a small caseload of patients who require longer-term support, typically those on waiting lists or who are too vulnerable to disengage with. Patients referred to the caseworker have minimal involvement with hospital-based services.  *Caseworker*  The caseworker offers high-intensity support for high-risk, high-need young people and engages with them two to three times a week. To maintain this level of contact, the caseload is limited to five young people at a time. Some service users may be on the caseload for an extended period if they resist engagement. However, for patients referred to hospital-based services, the caseworker has minimal involvement or contact. |
| **9. Tailoring** | *"If the intervention was planned to be personalised, titrated or adapted, then describe what, why, when, and how.*  **Level of harm**  The VPT engages with patients attending ED in consequence of their exposure to violence. All patients are eligible and initial assessment will determine how patients are managed. In the case of domestic abuse, the patient will be handed over to an Independent Domestic Violence Advocate (IDVA). In the case of sexual abuse, the patient will be handed over to the Independent Sexual Abuse Advocate or the Sexual Assault Referral Clinic. In the case of self-harm, the patients will be handed over Mental Health Services. The VPT will therefore typically engage with patients attending with non-domestic violence-related injuries, of varying acuity. The team offers immediate support to patients with non-life-threatening injuries, while for high acuity patients, they wait until the patient is stable before offering their services. The VPT team conducts assessments for patients admitted into the hospital, which allows them to provide more intensive support.  *Caseworker*  The caseworker engages with patients either in the hospital or in community settings and maintains a caseload of up to five young people at a time for high intensity support. |
| **Stage of Implementation** | *(i) What stage of implementation does the TIDieR checklist cover?*  *(ii) Is this a revision of an earlier TIDieR checklist*  This is an initial TIDieR, generic to two sites at which the VPT model has been implemented. The VPTs at the two sites are at different stages of implementation with one having a significantly longer operational period. |
| **How well - planned** | *“If intervention adherence or fidelity was assessed, describe how and by whom, and if any strategies were used to maintain or improve fidelity, describe them.*  The VPT model is subject to a formal evaluation, and a process and implementation evaluation, which this TIDieR informs. The outcome from these evaluations will be a revised logic model, an understanding of the adaptations made to the intervention based on locality, and a formal effectiveness and cost-effectiveness evaluation. |
| **How well - actual** | *"If intervention adherence or fidelity was assessed, describe the extent to which the intervention was delivered as planned.*  *Describe the extent to which the intervention was delivered as planned and outline the factors which had an*  *impact on actual delivery.*  Delivery of the intervention has been impacted by staffing changes in both sites, with both sites operating with reduced staff during periods of their operation. As a result, this has led to delays engaging with and referring some patients for further support. |
