# Supplementary material for: Effectiveness and Cost-Effectiveness of Emergency Department–Based Violence Intervention Programs in the United Kingdom: Protocol for a Quasi-Experimental Study
Source: JMIR Res Protoc. 2026 Feb 18;15:e86247. doi: 10.2196/86247 (PMC12961390; doi:10.2196/86247)
Supplement: Multimedia Appendix 4 [file resprot_v15i1e86247_app4.pdf]

Professor Simon Moore
Violence Research Group
School of Dentistry
Heath Park
Cardiff University
Cardiff, CF14 4XY

Ruth Swire
NETSCC Monitoring Team
National Institute for Health Research
Evaluation, Trials and Studies Coordinating Centre
University of Southampton
Alpha House, Enterprise Road
Southampton SO16 7NS

14 January 2022

Dear Ruth,

NIHR134055 - The Effectiveness and Cost-Effectiveness of a Clinical Violence Prevention Team Based in the Emergency Department

Thank you for your email, dated 10 January 2022, requesting additional clarification of our proposal. We have not made any substantive changes to the proposal, other than those detailed below. These further comments have allowed us to further strengthen the proposal.

With regards outcomes, we would like to highlight that the timeline includes a pilot analysis to inform the protocol development. This includes regular meetings with the Youth Endowment Foundation, the Home Office, and the police. It is probable that this work will identify opportunities to further enhance the project outcomes and, together with stakeholders, strengthen the protocol.

1) The outcome measures need further explanation, health utilisation (emergency department use) should be differentiated from health outcomes (reduction in emergency department attendance). This has not been addressed. The applicants have provided a list of the codes that are available that would help to describe the nature and details of health care utilisation (emergency department use). The point from the Board was that this is not the same as the outcome for the study - which is stated as a reduction in emergency department attendance. That would require description of how a reduction would be assessed - for example, a difference in the number of emergency department visits classed as violence or injury related, over 2 years following the index visit, between those attending intervention sites, compared to those in control sites. This needs to be properly specified.

We have added to the proposal:

The index attendance is an ED attendance classified as an assault-related injury. The outcome is any subsequent unscheduled reattendance into the ED. The outcome is compared across control and intervention sites as the rate unscheduled reattendance. In primary analyses we consider the Nelson Aalen cumulative hazard estimate across control and intervention sites and for the two years following the initial index attendance. This adaptation of the Andersen–Gill model allows us to derive the Hazard Ratio for subsequent ED attendance, comparing those in receipt of the intervention and those not in receipt of the intervention. The primary analysis will consider Hazard Ratios in Intention to Treat and Per-Protocol analyses. An advantage of the Anderson-Gill approach is that it can account for left-, interval (e.g., admitted into hospital) and right-side censoring and include baseline characteristics (e.g., age, gender, characteristic of the index attendance). We can further interact the intervention effect with time, to consider any non-linear intervention wane (usually specified as a negative exponential).

2) The proposed health economic methods were from an NHS perspective, rather than a societal perspective and should be refocused to be based on NICE public health economic evaluation methods recommendations. The applicants acknowledge this and say that they will "bring in evidence on the costs to the 3rd sector and to other parts of the public sector" - however this did not address the comments from the Board which asked for information in the protocol of HOW this will be done - what costs are available and where will they be obtained from? However, the point that the most robust data would relate to healthcare costs is taken, and that it makes sense therefore that an NHS perspective be used for the primary analysis.

Resource use from the NHS and public sector will be available from the ADR and SAIL databanks. Detailed healthcare resource use is available from SAIL including ED, outpatient and inpatient data which is akin to the Hospital Episode Statistics data available in England. Costs can be attached to these estimates of resource use based on NHS reference costs.

In addition to NHS costs, there are, broadly, three sources of additional costs that we seek to explore: (i) social care costs (e.g., children looked after by Local Authorities), (ii) other non-health statutory costs (e.g., police) and (iii) third-sector costs (e.g., homelessness charities).

i. SAIL data concerning resource use associated with social care and related activities (e.g., CAFCASS reports, court orders) is available and will be used to estimate the social care costs associated with intervention activities The Personal Social Services Research Unit (www.pssru.ac.uk) provides social care costs applicable to those who receive non-NHS support (e.g., adult or children's social worker). This will extend to, for example, victim services, court orders the unit costs of which are available (1).
ii. Resource use data will be available on crime type (e.g., assault with injury) and disposal methods (e.g., rehabilitation order, caution, custody) from ADR court data. Police costs are available by crime type (2) as are disposal costs (1), which we believe can be inferred from court data.
iii. Third sector resource use and costs are not routinely documented, and we are unlikely to be able to access individual specific resource use associated with any services received. However, via the VPT teams, we will have information about which individuals are referred into different 3rd sector organisations. We will then liaise with these organisations to estimate the typical average cost incurred of an individual referred to their services. For this we will use both bottom-up approaches, based on the development of detailed questionnaires to be completed by organisation staff, and top-

down approaches, based on total organisation costs and throughput, to estimate the average cost of an individual referred to them. For example, The Wallich homelessness charity had a total annual expenditure (2020) of £15.3M, supporting 9,664 individuals, at an estimated unit cost of £1,592 (see www.thewallich.com/app/uploads/2019/07/Support_that_Saves.pdf for further breakdown).

We have revised the Health Economic section as follows:

Health economic evaluation

For the third question (Q3), the value for money of the intervention will be determined over a two-year follow up period from the perspective of the NHS, with all NHS costs considered in the base case and health outcomes measured in quality-adjusted life-years (66). Other health care outcomes measures will also be considered (e.g., life years, ED attendances avoided). We will also explore value for money from wider perspectives including different parts of the public sector (e.g., reflecting costs and outcomes relevant to safeguarding, the police and education) (67-69). If differences are observed over this period that are expected to persist beyond two years, a decision analytic model will be developed to estimate value for money over the longer term. Resource use from the NHS and public sector will be available from the ADR and SAIL databanks. Detailed healthcare resource use is available from SAIL including ED, outpatient and inpatient data which is akin to the Hospital Episode Statistics data available in England. Costs can be attached to these estimates of resource use based on NHS reference costs. The analyses will be conducted for both the individuals eligible for VPT (primary analysis) and their households (secondary). An exploratory distributional cost-effectiveness analysis will be conducted to examine the impact of the intervention on the socioeconomic distribution of health (i.e. does the intervention benefit the socioeconomically deprived) (70).

The economic evaluation will consider the costs of delivering the intervention, and any impacts on downstream healthcare resource use. All health care resource use, including ED attendances, primary and secondary care resource, will be costed. For the two-year analysis, we will use the ADR and SAIL datasets to estimate the impact of the VPT model on resource use and outcomes over the study period. VPT set up and running costs will be examined by a standardised costing exercise (e.g., staffing levels, training, consumables, overheads) and from VPU commissioning documents (see further details below). We will explore costs more generally, with colleagues in the VPU the resource use on the police will be estimated. Costs falling on other sectors will also be considered where available. For example, costs resulting from available resource use on the police will be estimated. Given the potential for individuals to be referred to 3rd sector organisations, efforts will be made to estimate the costs of an average client interaction with these organisations (see further details below).

For the intervention costs, we will have estimates of salary costs and information on the number of contacts with the VPT team so will be able to estimate total and per contact costs for the intervention. Downstream healthcare resource use will be captured within the SAIL data (inpatient, outpatient, ED attendance, NHS substance misuse services, etc.). We will then attach appropriate unit costs to this resource use to estimate health care costs (e.g., NHS reference costs, PSSRU unit costs of health and social care).

In addition to NHS costs, there are, broadly, three sources of additional costs that we seek to explore: (i) social care costs (e.g., children looked after by Local Authorities), (ii) other non-health statutory costs (e.g., police) and (iii) third-sector costs (e.g., homelessness charities).

i. SAIL data concerning resource use associated with social care and related activities (e.g., CAFCASS reports, court orders) is available and will be used to estimate the social care costs associated with intervention activities The Personal Social Services Research Unit (www.pssru.ac.uk) provides social care costs applicable to those who receive non-NHS support (e.g., adult or children's social worker). This will extend to, for example, victim services, court orders the unit costs of which are available (80).
ii. Resource use data will be available on crime type (e.g., assault with injury) and disposal methods (e.g., rehabilitation order, caution, custody) from ADR court data. Police costs are available by crime type (73) as are disposal costs (80), which we believe can be inferred from court data.
iii. Third sector resource use and costs are not routinely documented, and we are unlikely to be able to access individual specific resource use associated with any services received. However, via the VPT teams, we will have information about which individuals are referred into different 3rd sector organisations. We will then liaise with these organisations to estimate the typical average cost incurred of an individual referred to their services. For this we will use both bottom-up approaches, based on the development of detailed questionnaires to be completed by organisation staff, and top-down approaches, based on total organisation costs and throughput, to estimate the average cost of an individual referred to them. For example, The Wallich homelessness charity had a total annual expenditure (2020) of £15.3M, supporting 9,664 individuals, at an estimated unit cost of £1,592 (see www.thewallich.com/app/uploads/2019/07/Support_that_Saves.pdf for further breakdown).

See Secondary Outcomes for further detail.

Health impacts on individuals will be measured in terms of life years and quality adjusted life years (QALYs), with QALYs estimated by capturing the health-related quality of life (HRQoL) decrements associated with ED attendances. We will have information available on the assault related injury, location of the injury, and severity (triage category, Glasgow Coma Scale). We will explore alternative approaches (and combinations thereof) to estimating the QALY impacts for assault. In the base case, we plan to take the following steps: (i) using injury categories, we will undertake targeted literature reviews to identify QALY and HRQL decrements (previous studies have estimated these for assault related injuries (72, 73)) and combine these with the available injury-QALY mapping available through AWIS and, (ii), apply those QALY decrements in the study; (iii). If necessary, we can revise the QALY decrements are using EQ-5D scores from published catalogues (74) with evidence on durations of injury and expected period of recovery (74, 75). (iv). We will undertake sensitivity analysis using alternative values. The All Wales Injury Surveillance System has also mapped injury codes in PEDW (ICD10), EDDS (ED Diagnostic Codes) and Cause of Death (ICD10) data to disability-adjusted life-years (DALYs) (see www.awiss.org.uk/all-cause-injuries-disability-adjusted-life-years; (71)) which will be considered as a scenario analysis.

Statistical methods for estimating the impact of the VPT model on costs and outcomes will replicate those of the statistical analyses (see above).

Cost-effectiveness will be assessed from a health care perspective in the base case, with cost-effectiveness assessed based on appropriate cost-effectiveness thresholds. Sector specific and aggregated net benefit decision rules will also be presented where possible, which reflect the benefits of an intervention less its opportunity costs in a given sector (i.e., the benefits which could be generated if the required resources were used for other purposes) (67, 76). Disaggregated results will also be reported using an extended impact inventory approach (69, 77). If the effects are believed to persist beyond the study period, a decision model will be developed to link any changes over the short term to longer term costs and consequences. We will also consider the cost-effectiveness of any national rollout (78, 79).

3) The noted secondary outcomes could be more comprehensive... The applicants do helpfully explain some of the limitations of the available datasets, but the response remains vague as to what other data is available which could allow analysis of secondary outcomes such as those related to criminal justice. The response states that there is data from CAFCAS and there will be information on police involvement - but this doesn't translate to what specific data and how these can relate to relevant outcomes. Please add in what secondary outcomes will be possible to examine, and which additional outcomes may be available if access to additional datasets allows. For example, apart from receipt of a PPN, would police data allow evaluation of any other outcomes related to criminal justice consequences?

We have revised the outcomes. We have dropped the eating disorder outcome, as the relationship with violence is unclear. We have emphasised the role measurable outcomes have in deterring offenders (e.g., prosecution) or protecting those who are vulnerable (e.g., safeguarding). In so doing, secondary outcomes identify additional health needs and activities aimed at preventing future harm attributable to violence and they are more theoretically plausible. Many thanks for the steer in this respect.

The court data referred to will require work to bring into the project. While we have expertise on the project team (TL is a magistrate, for example), it is not possible to state definitively what specific outcomes we are able to consider and whether there are sufficient numbers to allow their formal consideration and these data will most certainly overlap with CAFCASS and Looked After Children data. These data are complex, and this will be the first project to make use of them in an evaluation. We do have very strong links with the police forces in Wales and, understandably, the Violence Prevention Unit. And we have previously worked with both police data and similar (e.g., Home Office Offenders Index data). We remain, however, reluctant to over-promise. The call was to evaluate police-related activities that influence health outcomes, which we address, and both the project team and wider stakeholder groups are enthusiastic about the opportunities to consider criminal justice activities more broadly.

We have revised the proposal:

There is ongoing work in ADR Wales to link Ministry of Justice police data. We cannot state definitively that these data will be available to the current project, however we do believe that should it become available then we will have information on police disposal codes (e.g., caution, custody) that can be linked with ED data. ADR UK do have Data First magistrates' court defendant

data catalogue, Data First Crown Court defendant data catalogue, Data First criminal courts and prisons linking data catalogue, Data First prisoner custodial journey data catalogue, Data First Family Court data catalogue, including domestic violence remedies, and outcomes relating to the Children Act.

Social care activities are available across GP data, the Children in Need dataset, and CAFCASS data and will include emergency protection orders, care orders, supervision orders, and placement orders (3). This involves CAFCASS officers checking criminal records and police files, as well as seeing if the family are already known to social services. They will also contact all the adults in the case and ask if they have any concerns over the safety or welfare of the children. CAFCASS reports to the courts. There are occasionally instances where agreement between, for example, a Local Authority and parents, outside of the court and therefore the CAFCAS process. In which case, details of looked after children would appear in the Children Receiving Care and Support census. However, details of vulnerability, safeguarding and looked after children are also available in GP data.

In sum, “Criminal Justice” is a broad term, one covering public and family law, and several agencies including the police, social care and the courts. This system should work in harmony to protect those who are vulnerable and deter offenders. Competencies vary, but ultimately the courts will determine any formal sanction on an offender and any safeguarding measures. However, only a proportion of cases will ascend to the courts, and much of the work undertaken will occur through MARAC or similar. In which case, CAFCASS and PPNs will provide additional information.

- Additional health outcomes
    - Any referrals from ED into secondary care
        - The Outpatient Referral dataset captures referrals across the health estate in Wales, therefore facilitating referral activity comparisons across cases and controls. These data capture referral from ED to other specialities, but not the reasons for referral. Given the assumption that the VPT represents the only opportunity for some to access healthcare, we can capture case and control referral activity, which we would expect to increase for case patients.
        - Outcome: a comparison of total referrals from ED for case and control patients
    - Mental Health
        - From the GP data set, evidence of a common mental disorder (CMD) can be recovered (57). The use of GP data provides an opportunity to evaluate the likelihood that a participant was experiencing a CMD using data that does not rely on self-report. Participants’ CMD is derived algorithmically and using the presence of an historical or current diagnosis, depressive or anxiety symptoms and current anti-depressive or anti-anxiety treatment plus current untreated diagnosis. The algorithm is run on over a six-month window. We assume that the absence of symptoms, treatment or diagnosis in the six months preceding a positive case would be indicative of remission. The analytic date for CMD status is defined as the mid-point of the six-month window. Each window is repeated every three months to allow complete coverage and to identify change in CMD status and therefore remission and relapse.
        - Outcome: mental health diagnosis in case and control patients.
    - Substance Misuse

        - Presentation for substance misuse treatment in Wales. These data capture whether patients have been referred into treatment, the substance of abuse, including alcohol, admission, and discharge dates.
        - Outcome: engagement with substance misuse treatment services for case and control patients.

- Young people
    - Educational outcomes (16 years and younger)
        - The effect on children can include poor key stage educational outcomes, which is in turn related to later life health outcomes (55). These data capture attainment, absenteeism (authorised and unauthorised), and exclusions (56).
        - Outcome: key stage educational attainment in case and control children; and key stage educational attainment of children in case and control households.

- Prevention
    - Public Protection Notices
        - Receipt of a Police Public Protection Notice (PPN) in which the participant is named, irrespective of whether they are perpetrator or victim. Police officers identify adults at risk (Care Act 2014) and PPNs are sent to Safeguarding Referral Units with the expectation that the local authority adult services consider a multi-agency adult safeguarding enquiry (58). We hypothesise that the VPT increases ascertainment of ARA and as the intervention Standard Operating Procedure requires referral to the police for stabbings, and general advice to all patients to contact the police, we anticipate that there will be an increase in PPNs issued for case participants, compared to controls. An ongoing project (Brophy: NIHR133680) has incorporated Welsh police force data into SAIL and linked with this ED data. These data indicate that 31% of men and 34% of women in receipt of a PPN also have one or more emergency healthcare attendances.
        - Outcomes: Whether the attending officer submitted a PPN form due to a safeguarding concern for requirement for additional support from partner agencies.
    - Ministry of Justice: Data First
        - ADR UK have Data First magistrates' court defendant data catalogue, Data First Crown Court defendant data catalogue, Data First criminal courts and prisons linking data catalogue, Data First prisoner custodial journey data catalogue, Data First Family Court data catalogue available for linkage. From these data, we can determine whether an order weas made to protect an individual or individuals and the result code for those charged with violence against the person.
        - Outcomes: whether the perpetrator was formally charged, orders on offenders in respect of sanctions or rehabilitation. Any safeguarding order.
    - Safeguarding
        - Social care activities are available across GP data (e.g., SCTID: 1010196001), the Children in Need dataset, and CAFCASS data and will include emergency protection orders, care orders, supervision orders, and placement orders. This involves CAFCASS officers checking criminal records and police files, as

well as seeing if the family are already known by social services. They will also contact all the adults in the case and ask if they have any concerns over the safety or welfare of the children. After these initial enquiries, CAFCASS will provide a short letter to the courts which is known as the 'safeguarding letter'. There are instances where agreement between, for example, a Local Authority and parents, occurs outside of the court and therefore the CAFCAS process. In which case, details of looked after children would appear in the Children Receiving Care and Support data.

- Outcome: safeguarding

4) Intervention costs. The response is confusing. This point related to the methods for the economic evaluation - but the response seems to be partly around study intervention costs. In the last part of the response, the applicants seem to have partially responded to the point. However, it still lacks detail. Please specify the sources that will be used for estimating costs. For example, in the health economic analysis section it states: "All health care resource use, including ED attendances, primary and secondary care resource, will be costed". This lacks detail on what source(s) will be used to estimate these costs. Similarly, what data will be used to estimate "resource use on the police" or "the costs of an average client interaction".

Please see the response to point two above in which we specify resources available to cost activities across the NHS, and social care.

5) PPI - please specify the specific aspects that will be developed further with PPI involvement. For example, to prioritise the secondary outcomes, or to refine costing etc.

We have added to the proposal:

i. In addition to commenting on the conduct, methods, and outcomes of the evaluation, one of the opportunities in this project is clarifying how those who experience violence might seek assistance and support. Our primary interest focuses on the Emergency Department, beyond this it is feasible that there are many other services that could have offered support, had patients been aware, or were contacted but were unable to provide support. Given the mixed experiences of the PPI groups we will consult, we aim to ensure our analysis of patient pathways is consistent with real-world experience and in this sense generate narratives that provide an illustration of the quantitative results.

ii. We will endeavour to prioritise, or rank, secondary outcomes with PPI groups in order that the most bring forward the most salient outcomes and explore opportunities for further inclusion of data is warranted.

iii. Building on i and ii, we will ask PPI groups to consider the costs estimated for patients and, with their assistance, seek to develop a narrative account of the costs involved with violence. While we can capture the tangible costs associated with healthcare, intangible costs, for example to

individuals, families, and communities, will be significant and should be represented, albeit in narrative form.

Thank you for the additional feedback, and if there are further matters arising, please do get in touch.

Yours,

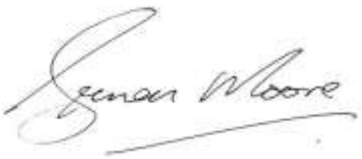

Professor Simon Moore

References

1. Newton A, May X, Eames S, Ahmad M. Economic and social costs of reoffending: Analytical report. London: Ministry of Justice; 2019.
2. Heeks M, Reed S, Tafsiri M, Prince S. The economic and social costs of crime: Second edition. Home Office Research report 99. 2018.
3. Bedston S, Pearson R, Jay MA, Broadhurst K, Gilbert R, Wijlaars L. Data resource: Children and Family Court Advisory and Support Service (Cafcass) public family law administrative records in England. International Journal of Population Data Science. 2020.
