# Supplementary material for: Effectiveness and Cost-Effectiveness of Emergency Department–Based Violence Intervention Programs in the United Kingdom: Protocol for a Quasi-Experimental Study
Source: JMIR Res Protoc. 2026 Feb 18;15:e86247. doi: 10.2196/86247 (PMC12961390; doi:10.2196/86247)
Supplement: Multimedia Appendix 2 [file resprot_v15i1e86247_app2.pdf]

NIHR Evaluation, Trials and Studies Coordinating Centre (NETSCC)
University of Southampton
Alpha House, Enterprise Road
Southampton, SO16 7NS

Email: phr@nihr.ac.uk

25 June 2021

Professor Simon Moore
Violence & Society Research Group
Cardiff University
University of Cardiff
Cardiff
CF14 4XY

Dear Professor Moore,

NIHR134055 - The Effectiveness and Cost-Effectiveness of a Clinical Violence Prevention Team Based in the Emergency Department

Your Stage 1 application was considered by the June PHR Funding Committee. I am pleased to inform you that the Funding Committee wishes to invite you to submit a Stage 2 application for consideration at its next meeting in October 2021.

The committee membership is made up of senior, experienced researchers, methodologists (including triallists, statisticians and health economists) and public contributors, who are all active in their field. When assessing proposals, the committee considers how well they fulfil the criteria of scientific quality, feasibility and value for money.

The Funding Committee would like you to consider the following comments when preparing your Stage 2 application. Please note that these are not in order of importance.

1. The committee thought this was an ambitious proposal focussed on an important issue and interesting intervention.

2. However, the application lacked detail throughout. The aims and objectives were too vague and the study design and methodology needs to be clearly described and specified.
3. The outcome measures need to be further explored. The use of the health service should be differentiated from health outcomes. The wider societal impacts should also be explored further.
4. Reassurance is needed around the feasibility of obtaining data to inform the health economic evaluation. Please specify how and where the data will be sourced.
5. The team may wish to consider propensity score matching the intervention to the control group. The uniqueness of Cardiff, and differences between Cardiff and the rest of Wales, need to be unpicked.
6. It is noted that the process evaluation is being conducted by PH Wales. It would be helpful if this work could be formally linked to this study and the relationship and interdependencies further described.
7. While most PPI costs had been included, the PPI lead has 0% fte; this should be amended. It was positive to see the involvement of a homeless support charity. The team should expand this to include other charities/groups that can help represent the target groups.
8. The plain English summary was well written.
9. The dissemination plan needs to be further fleshed out.
10. Applicants are encouraged to engage with their local Clinical Research Networks when developing their Stage 2 applications.

Accessing your Stage 2 application form
Please log into your user account for the NETSCC Management Information System at this URL link to complete the Stage 2 application: https://netscc-mis.nihr.ac.uk/mis. The Stage 2 application task will be available under 'My Tasks'. Please ensure you refer in detail to the Stage 2 guidance notes and supporting information as they contain the essential information to successfully complete your form.

Please note that these application guidance notes have recently been amended to include important NIHR changes and updates which applicants need to take note of and reflect in their stage 2 applications. The changes/updates include the following:

- More explicit guidance for applicants on NIHR's expectations with regard to patient and public involvement
- New guidance for applicants on Equality, Diversity and Inclusion for study participants (research following patient need and clusters of morbidity)
- New guidance information about the provision of applicants' equality and diversity monitoring information

- A broadening of the guidance wording to ensure appropriateness for social care research

If you have any difficulties or would like clarification on any points please send your queries to me via email: phr@nihr.ac.uk

Completing your Stage 2 application
To save you time, some details from your Stage 1 proposal have been saved and inserted into the Stage 2 proposal form. It is important that you check that this information is correct before adding further details and submitting your application. Any information that has been automatically added to the Stage 2 form from your Stage 1 proposal should be considered in light of the above feedback and revised as necessary.

Your Stage 2 application and required uploads **must be submitted** electronically using the 'Submit' button on the last page of the web form, 'review and submit', by **1pm on 20 August 2021.** Please note that failure to fully comply with these instructions may result in your application being declined.

Due to current circumstances, please contact us if you are unable to meet the deadline provided and we will consider deferring your submission. Any deferral is likely to be for 4 months; we will advise you of the new Stage 2 deadline.

External peer review
The main sections of the Stage 2 application are subject to external peer review and lead applicants will be invited to submit a response to anonymised reviewer comments ahead of the October Funding Committee. We will aim to send you these during the week commencing 6 September 2021 and you will have two weeks to return your responses. As some reviewer comments are not received until shortly before the committee meeting it may not be possible to seek an applicant response to these comments and the committee will be informed of this.

Co-applicants and signatories
As part of completing the form, you will need to request that your co-applicants and signatories complete their acceptance and collaborate tasks online. You will not be able to submit the form without their involvement so please factor the necessary time into your application planning. Also, please ensure you have provided all the required uploaded documents specified in the guidance and as part of the Stage 2 application.

Costs
Applicants should note that it is in their interests to undertake a thorough, realistic and accurate costing of their proposal. The PHR Programme accepts that some variance in costs is likely to occur between the Stage 1 application and the Stage 2 application. The Funding Committee will carefully scrutinise all Stage 2 costs and any variance from the initial stage. All costs need to be fully justified by the applicants to reassure that the study offers good value for money.

Before attempting to complete the online form, please read the 'Detailed Budget' section of the Stage 2 Guidance notes, this section includes guidance on the **Schedule of Events Cost Attribution Tool (SoECAT)**. For more advice on NHS costs and funding, please contact the Finance Manager at NETSCC (e-mail: nets-finance@nihr.ac.uk).

Compliance with the NIHR policy on clinical trial registration and disclosure of results
For all primary research studies, please ensure that you provide the information requested in the Research Grants held section of the Stage 2 guidance notes. Retrospective study registration is encouraged, and a reason for non-registration or non-publication should be provided where applicable.

Study Commencement
Please plan to **start your project on the 1st of a month** and give a realistic start date to allow for staff recruitment etc., bearing in mind that Stage 2 applications will be considered by the Funding Committee in **October 2021** and some revision may be required subsequently. Please note that projects are expected to start within eight months of the date of the final funding outcome letter.

The programme is interested in taking advantage of the growing utility of routine data. Where appropriate we would like investigators to ask study participants to consent to long term follow up (e.g. beyond the outcomes to be collected in the funded study) using routinely collected data, and appropriate linkage to allow this data to be best used.

I would be grateful if you could please email me by **one week from the date of this letter** to confirm your intention to submit a Stage 2 proposal. If you have any queries please contact us at phr@nihr.ac.uk.

Yours sincerely,

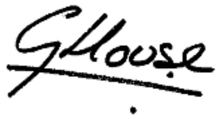

Gemma House
Senior Research Manager
PHR Programme
