# Supplementary material for: Effectiveness and Cost-Effectiveness of Emergency Department–Based Violence Intervention Programs in the United Kingdom: Protocol for a Quasi-Experimental Study
Source: JMIR Res Protoc. 2026 Feb 18;15:e86247. doi: 10.2196/86247 (PMC12961390; doi:10.2196/86247)
Supplement: Multimedia Appendix 3 [file resprot_v15i1e86247_app3.pdf]

NIHR | National Institute for Health Research

NIHR Evaluation, Trials and Studies
Coordinating Centre (NETSCC)
University of Southampton
Alpha House, Enterprise Road
Southampton, SO16 7NS

Email: phr@nihr.ac.uk

11 November 2021

Professor Simon Moore
Violence & Society Research Group
University of Cardiff
Cardiff
South Glamorgan
CF14 4XY

Dear Professor Moore,

NIHR PHR Reference Number: NIHR134055 - The Effectiveness and Cost-Effectiveness of a Clinical Violence Prevention Team Based in the Emergency Department

I am pleased to inform you that the PHR Funding Committee has recommended your application submitted for consideration in **20/119 Public health interventions led by or involving the police** for funding and the Department of Health and Social Care, in their capacity as the National Institute for Health Research (NIHR), has confirmed their intention to award funding upon acceptance of the terms and conditions set out in the Standard Research Contract and pending agreement to the suggested amendments recommended by the Funding Committee, as detailed in the enclosures.

The Standard Research Contract, between Contractors and the Secretary of State for Health and Social Care for all initiatives can be found on the NIHR website at:

https://www.nihr.ac.uk/researchers/apply-for-funding/how-to-apply-for-project-funding/sign-a-contract.htm.

Next Steps

The NIHR is committed to the rapid initiation of research following the decision to fund to benefit members of the public and patients as soon as possible. Therefore, we expect funded researchers to be working towards gaining the necessary contractual agreements and governance approvals required to start the project within 8 months or by a date mutually agreed by both parties on acceptance of the award.

The NIHR acknowledges the risk to organisations around committing resource to research before a contract is in place; however, it is rare to not reach contractual

terms unless the circumstance of the research team changes. The NIHR, therefore, encourages organisations to commit staff to setting up projects at as early an opportunity as possible in order to expedite the formal commencement of research.

It is acknowledged that there can be unforeseen delays in starting up a research project, but in order to help reduce these it is your responsibility to work closely with your organisation's R&D department or equivalent as well as other colleagues/departments involved in the administration and management of the research, and to start these discussions at the earliest opportunity.

To ensure that the project starts within the agreed timeframe with all the required agreements and approvals in place, appropriate staff (such as project and/or study managers) need to be in post as early as possible after receiving this letter of intent. These staff costs will ultimately be covered through the research funding award, but you are encouraged to meet them from Research Capability Funding (RCF) or equivalent prior to the research contract being agreed.

To support the often-iterative process towards agreement of the contract, we have set out the timeframes for the submission of responses or information for each step towards the agreement of the Standard Research Contract as well as the anticipated start date.

- Responses to Funding Committee feedback and queries – Four weeks from the date this email notification was sent.
- Responses to Finance and IP queries – Four weeks from the date this email notification was sent.
- Contract signature – no later than 7 months.
- Contracted commencement start – no later than 8 months or a date mutually agreed on acceptance of award.

On receipt of information as set out above, the NIHR through NETSCC is committed to responding to your submission of information within two weeks or we will update you on progress.

The enclosures to this letter detail the feedback on your application, the processes to be undertaken during the next steps, and additional information relating to your award. Your contact person within NETSCC is Ruth Swire, who will be working with you on the contract.

Yours sincerely,

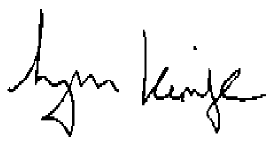

Lynn Kerridge
Chief Executive Officer, NETSCC

Enclosures:

- Funding Committee, Finance and IP Feedback
- Research Management and Approvals Information

cc: Mr Chris Shaw
Cardiff University
ShawC3@Cardiff.ac.uk

ENCLOSURE 1 – Comments and Feedback

Contact Details and Next Steps:

We understand that, in order for new studies to begin recruiting to their research team, details will need to be placed in the public domain (e.g. on websites) and discussions held with organisations supporting or involved in the study. However, aside from activities relating to recruitment of staff or project start-up we ask that you do not actively publicise or promote the award until the Research Contract has been fully signed.

Please complete your revised application, with a letter of response and a revised detailed project description no later than **1pm on 9th December 2021.**

Going forward your contact person is Ruth Swire, who can be contacted on netspostawardsetup@nihr.ac.uk.

Please log into your user account for the NETSCC Management Information System (NETSCC MIS) at https://netscc-mis.nihr.ac.uk to respond to the comments and requirements for changes. You will have access to the relevant parts of your application form to revise and resubmit with the required changes. Please note it is expected that this will be the final version of your application form, so please ensure that all justifiable relevant costs are included where appropriate.

Additionally, as part of the submission, you should include a letter that responds to each comment point in turn and clearly explains your proposed action(s). If changes to your detailed research plan are required, you should also provide a revised copy of your plan which shows all the amendments as 'tracked changes' and which includes a version number and date. Please also upload a 'clean' copy of the plan with all tracked changes accepted.

These documents should be included as PDF uploads when submitting your revised application. Please note that you should delete any old versions of the detailed research plan currently showing on the MIS before uploading the revised versions.

Please note: Now that your project has been recommended for funding (subject to a satisfactory response to the points raised in your intent to fund letter) we would like some additional details to help us monitor partnerships with CTUs and help us understand the work leading up to this project. The revised application has two additional pages titled 'Application Background' and 'Clinical Trials Units'. Please complete these additional pages before submitting your revised application.

Funding Committee Feedback

1. The committee acknowledged the importance of research in this area and that although some feedback/comments had been well addressed, others were not adequately addressed; such that key methodological details and outcome measures lacked sufficient detail.

2. The outcome measures need further explanation, health utilisation (emergency department use) should be differentiated from health outcomes (reduction in emergency department attendance).
3. Further reassurance is required around the feasibility of obtaining data to inform the health economic evaluation. Can the type of harm be obtained from the linked data?
4. Health economic evaluation was still lacking in detail, there was little granularity around how QALYs will be estimated.
5. It was considered that there was a mismatch between the health economic evaluation objectives and the proposed methodology, specifically the details linking the routine data obtained and whether this matched up with the categories was not alluded to in the response nor in the stage 2 health economics text.
6. The proposed health economic methods were from an NHS perspective, rather than a societal perspective and should be refocused to be based on NICE public health economic evaluation methods recommendations (see: https://www.nice.org.uk/process/pmg4/chapter/incorporating-health-economics
7. The potential unintended consequences of the intervention had not been addressed adequately. Applicants should provide further details on how they will consider whether the proposed intervention could deter individuals from attending the emergency department in the future.
8. The noted secondary outcomes could be more comprehensive, for example they do not include safeguarding outcomes, criminal justice involvement, and employment / trainin. The applicants respond to the safeguarding outcomes but do not answer the question in relation to criminal justice involvement, which is high cost. This is unexpected given it is specifically referred to in the application set up … 'There are aspects of support that can be provided by healthcare, and criminal justice will also play a significant role, particularly in challenging offenders.
9. More details on the the costs of delivering the intervention are needed, and any impacts on downstream healthcare resource use. Specific details are needed on how these will be identified, measured and valued. There are lots of mentions of 'costs being considered/explored' but no details on how.
10. Further information on how sample size was calculated is still required.
11. Public involvement was strengthened in response to Stage 1 feedback. These new links should be used when addressing some of the committee and reviewer feedback during the lifespan of the project.
12. The plain English summary requires some rewriting making the language more accessible.
13. Further clarification on the role of the focus groups is required and whether these are part of the research, or PPI.
14. Reassurance is required that funding for the Cardiff site will continue throughout the study period. If this is not the case then please provide information regarding the potential impact this may have on the study and related mitigation strategies.

15. Applicants should provide details on their plans for ethical considerations of the application; safeguarding of vulnerable individuals, any criminal justice involvement and employment training for a vulnerable population.
16. One Postdoctoral Researcher (100%) is included for project management, dissemination, literature review, data collection, support to analysis and health economics (e.g. third sector costs). To have one RA who is able to cover all these roles is unusual – can this be justified more fully.

Finance Feedback

1. Management group meetings costs for travel and subsistence seems quite high, please consider if any of these could take place virtually to reduce costs.
2. Where costs are included for two International Conferences please confirm the number of attendees to each (it is noted in the justification of costs that this is for two but it is not clear whether that is two to each, or one to each). Please note we permit a maximum of one attendee to two international conferences, or two attendees to one international conference.
3. Please provide a full breakdown of costs included for University of York consumables - these should be itemised on the budget and be project specific.
4. Where costs are estimated for a Police Research Practitioner please provide more information on how this has been estimated e.g. number of days/hours and rate used or FTE if salaried.
5. Annual salaries costs are missing for the following staff - please update the budget with annual salary, Superann. and Nat. Insurance:
    - Brophy, Sinead
    - Watkins, Alan
    - David O'Reilly
    - Swansea Research Assistant (Number 4 in list)
6. Where costs have been included as FTE costs for PPI Co-app Yeomans, Henry and Battaglia, Adele please advise if these have been calculated on a PPI rate/consultant basis rather than a salaried FTE cost? If so please move these costs to the PPI cost section and provide a rate used for each. If these are to be employed roles please update the annual salary costs to include basic salary, Superann. and Nat. Insurance for both.
7. For all staff members listed as TBA please identify in both the annual costs of salaries section and the FTE costs as TBA 1, TBA 2, TBA 3 etc for clarity and reconciliation purposes.
8. FTE costs are missing for Shepherd, Jonathan please may these be included or advise if costs are not being claimed.
9. Please ensure that your annual budget profile reflects the likely activity in any given year.
10. Please ensure you include costs for translating study materials into other relevant languages, and any other elements which will ensure your study reaches all members of the potential participant community and other stakeholders.

If you require any additional advice about the attribution of costs, please contact the finance team, email nets-finance@nihr.ac.uk. Please also refer to the AcoRD guidance available at: https://www.gov.uk/government/publications/guidance-on-attributing-the-costs-of-health-and-social-care-research.

Intellectual Property (IP) Feedback

You are required to respond to all of these points in your letter as part of your response to the Funding Committee feedback.

1. If any third party owns or controls rights in the Background IP for the Research (for example if the Background IP is being used under licence), please consider the warranty given at clause 23.1.5(a) and the opportunity to list exclusions from this warranty at Schedule B of the research contract. If necessary, please complete Schedule B for our review and approval. Please note the Contractor need only complete Schedule B where it cannot warrant that it has an 'unrestricted and free right to use' the Contractor Background IP for the purposes of the Research. If there are no third-party rights, please confirm this in your response.
2. It is NIHR's starting position that all arising Foreground IP and Research Data shall vest with the Contractor. If you wish NIHR to consider alternative ownership arrangements then please provide your proposed wording so it can be reviewed and, if approved, will replace the standard wording to Schedule B.
3. If you have not already done so, I would strongly encourage you to discuss the above points with the appropriate department at your University/ Trust e.g., Research Office, Technology Transfer Office, Contracts Office etc. Please provide me with the contact details for your contact(s) in these departments so that I can copy them into future correspondence when appropriate.
4. This study will produce data, which is IP, and which may inform further studies. Although this may have no commercial value there is potential public benefit. The applicants must consider what foreground IP will be generated and how this will be managed to maximise benefit.
5. Please provide copies of any draft collaboration agreements relating to this study.
6. Please clarify the background IP in the 'dissemination, outputs and impacts' section of the Detailed Research Plan.
7. Confirmation that licences are either not required, or have been sought, for access to data relating to this study, should be provided.

Compliance with NIHR's Preventing Harm in Research Policy

Preventing harm in research is an overarching term that encompasses the policies and interventions put in place to protect individuals and address issues such as bullying and harassment or safeguarding. NIHR has an overarching policy document outlining its stance on these issues.

NIHR is committed to preventing and addressing any sexual exploitation, abuse, or harassment of research participants, communities and research staff, plus any

broader forms of violence, exploitation and abuse, such as bullying psychological abuse and physical violence. To meet this aim NIHR encourages research institutions who have not already done so to put in place a safeguarding policy whose principles comply with the NIHR safeguarding guidance. This is a contractual requirement from April 2021.

In your response letter, please will you confirm that your host institution has a safeguarding policy in place whose principles comply with the NIHR Safeguarding Guidance.

ENCLOSURE 2 – Research Management and Approvals Information

Research Governance

Guidance on the application process for ethical and other approvals can be found on the HRA website. Please note that if your study is led from England and involves the NHS in England you should apply for HRA approval.

If you are using patient information from an existing database, you should check whether the patients have given their consent for their data to be included in that database for research purposes, or if not whether the database is exempt under Section 251 of the NHS Act 2006. Where exemptions are not already in place, approval to use confidential patient information without consent must be requested from the HRA who make decisions with advice from the Confidentiality Advisory Group (CAG).

To take advantage of the growing utility of routine data (such as HES, GP records etc.), we would encourage investigators, where appropriate, to ask study participants to consent to long term follow up (e.g. beyond the outcomes to be collected in the funded trial) using routinely collected data, and appropriate linkage to allow this data to be best used.

Information on the responsibilities of key research stakeholders including Sponsors, Chief Investigators and Research Teams are summarised in the UK policy framework for health and social care research.

Clinical Research Networks

NETSCC is a National Institute for Health Research (NIHR) non-commercial Partner. This means the studies that we fund may be eligible to access NIHR Clinical Research Network (CRN) support, which is provided to health and social care research teams both in and outside the NHS. Where appropriate we expect you to apply for NIHR CRN support and subsequent inclusion in the NIHR CRN Portfolio of studies to fully benefit from the support that the CRN offers through their Study Support Service. To find out more, please visit www.supportmystudy.nihr.ac.uk. If your study involves NHS sites in England you will need to apply for Health Research Authority Approval. For guidance on submitting an application please visit: www.hra.nhs.uk.

Clinical Trials Authorisation

You may require a Clinical Trial Authorisation from the Medicine and Healthcare products Regulatory Agency (MHRA). You should contact the MHRA and begin the application process. The MHRA website (www.mhra.gov.uk) has the latest up to date supporting information and guidance. The DHSC/MRC website (www.ct-toolkit.ac.uk) also contains the latest information about Clinical Trials regulations and a helpful FAQ page.

Clinical Trials Regulation

Trials involving medicinal products must comply with 'The Medicines for Human Use (Clinical Trials) Regulations 2004'. You have indicated on your full proposal that your sponsor will be Cardiff University and we will assume that this is still the case unless we hear from you. The Department of Health and Social Care find this nomination acceptable but they reserve the right to refuse this nomination at a later date if they are not satisfied with the arrangements put in place to conduct the trial.
